# Metabolic profiling of metformin treatment for low-level Pb-induced nephrotoxicity in rat urine

**DOI:** 10.1038/s41598-018-32501-3

**Published:** 2018-10-01

**Authors:** Yu-Shen Huang, Shwu-Huey Wang, Shih-Ming Chen, Jen-Ai Lee

**Affiliations:** 1 School of Pharmacy, College of Pharmacy, Taipei Medical University, 250 Wuxing St., Taipei, Taiwan; 2 Core Facility Center, Department of Research Development, Taipei Medical University, 250 Wuxing St., Taipei, Taiwan; 3 Department of Biochemistry and Molecular Cell Biology, School of Medicine, Taipei Medical University, 250 Wuxing St., Taipei, Taiwan

## Abstract

Chronic kidney disease is a worldwide problem, and Pb contamination is a potential risk factor. Since current biomarkers are not sensitive for the diagnosis of Pb-induced nephrotoxicity, novel biomarkers are needed. Metformin has both hypoglycaemic effects and reno-protection ability. However, its mechanism of action is unknown. We aimed to discover the early biomarkers for the diagnosis of low-level Pb-induced nephrotoxicity and understand the mechanism of reno-protection of metformin. Male Wistar rats were randomly divided into control, Pb, Pb + ML, Pb + MH and MH groups. Pb (250 ppm) was given daily via drinking water. Metformin (50 or 100 mg/kg/d) was orally administered. Urine was analysed by nuclear magnetic resonance (NMR)-based metabolomics coupled with multivariate statistical analysis, and potential biomarkers were subsequently quantified. The results showed that Pb-induced nephrotoxicity was closely correlated with the elevation of 5-aminolevulinic acid, d-lactate and guanidinoacetic acid in urine. After co-treatment with metformin, 5-aminolevulinic acid and d-lactate were decreased. This is the first demonstration that urinary 5-aminolevulinic acid, d-lactate and guanidinoacetic acid could be early biomarkers of low-level Pb-induced nephrotoxicity in rats. The reno-protection of metformin might be attributable to the reduction of d-lactate excretion.

## Introduction

Chronic kidney disease (CKD) is becoming more common^1^, influencing 10% of the human popultion^2^. Lead (Pb) is one of the pathogens leading to CKD^3^. The result from National Health and Nutrition Examination Surveys showed that the prevalence of CKD was ranged from 1.8–8.1% for adults in the elevation of blood lead (Pb) levels (BLLs) (<1.06 to >2.47 μg/dL)^4^. Although the toxicity patterns change with reduced exposure, the relation between Pb and kidney functions has repeatedly been documented. In 2013, the prevalence rate of BLLs ≥10 μg/dL and ≥25 μg/dL were 20.4 and 5.2 adults per 100,000 employed population in USA, respectively^5^. There is growing data suggesting that exposure to low-level Pb may be a risk factor for the development and the progression of CKD^6^. Several studies from Taiwan have reported low-level environmental Pb exposure as a risk factor for the progression of CKD^7–9^. Therefore, Pb-induced nephrotoxicity has been brought to public attention and cannot be underestimated^10^.

Current biomarkers are not efficient at detecting Pb-induced nephrotoxicity. In occupational Pb exposure, serum creatinine and blood urea nitrogen (BUN) are not suitable for evaluating renal dysfunction^11,12^. Our previous study demonstrated that rats with low-level Pb-induced nephrotoxicity had higher renal methylglyoxal and urinary d-lactate without increasing serum creatinine or BUN^13^. The elevation of serum creatinine and BUN may be advanced and not fully reversible in renal injury^14^. Therefore, the discovery of early biomarkers of low-level Pb-induced renal injury is necessary and important.

Metformin is one of the most commonly prescribed drugs for type 2 diabetes mellitus, and has pleiotropic functions owing to its activation of adenosine monophosphate-activated protein kinase (AMPK)^15,16^. Metformin had been contraindicated in patients with renal sufficiency^17^. However, our previous studies showed that it could attenuate renal injury via reduction of renal methylglyoxal^13,18,19^. In 2016, the U.S. Food and Drug Administration revised restrictions on metformin use in kidney impairment^20^. Consequently, metformin is a promising drug for renal injury, although its mechanism of reno-protection remains to be clarified.

Metabolomics is a fast-growing field and plays an important role in developing disease-related biomarkers. Non-targeted and targeted strategies are commonly used for metabolomics analyses. The former are suitable for discovering unknown biomarkers, and the latter are used to quantify well-known targets^21,22^. One human serum metabolomics study showed that co-exposure to heavy metals disturbs lipid and amino acid metabolism^23^. Inhalation of Pb-containing PM_2.5_ particles mainly affects metabolic pathways such as amino acid metabolism, TCA cycle and nitrogen metabolism in rat urine and serum^24^. In this regard, the interruption of amino acid metabolism is commonly found in heavy metal-induced toxicity^25^. However, other mechanisms are of concern. It is important to note that the identification of metabolites might be misleading, depending on the database; hence, quantification of potential biomarkers is important in non-targeted metabolomics^26^.

**Figure 1:**
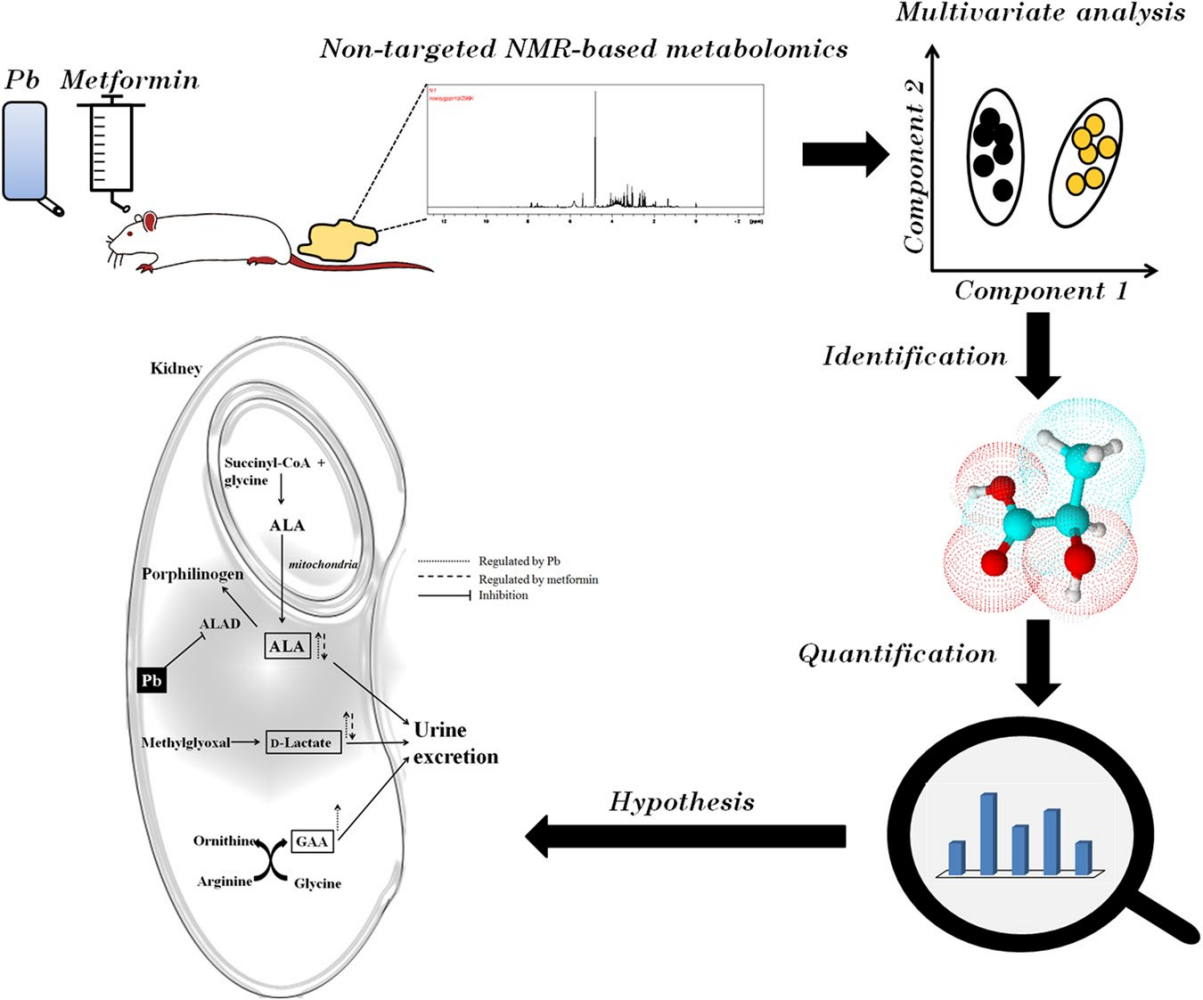
Study flow chart. First, the animal experiment was carried out, and urine was collected followed by NMR-based metabolomics. Second, NMR spectra were integrated into peak lists followed by multivariate analysis. Third, altered peaks were identified through MetaboHunter or MetaboMiner. Fourth, potential biomarkers were quantified through the HPLC or the LC/MS/MS method. Fifth, the hypothesis was showed according to our results.

To our knowledge, there is no evidence directly correlating the metabolic profile in urine with exposure to Pb and/or co-treatment with metformin. Therefore, we wanted to investigate metformin’s effect on the metabolic profile of rats suffering from Pb-induced nephrotoxicity using urinary metabolomics. Study flow chart was showed in Fig. 1. To confirm the results from metabolomics, quantitative methods were applied to measure the potential biomarkers. Our study aims to explain the metformin-related renoprotection, and discover potential biomarkers for early diagnosis of low-level Pb-induced renal injury.

## Results

### Histological examination

**Figure 2:**
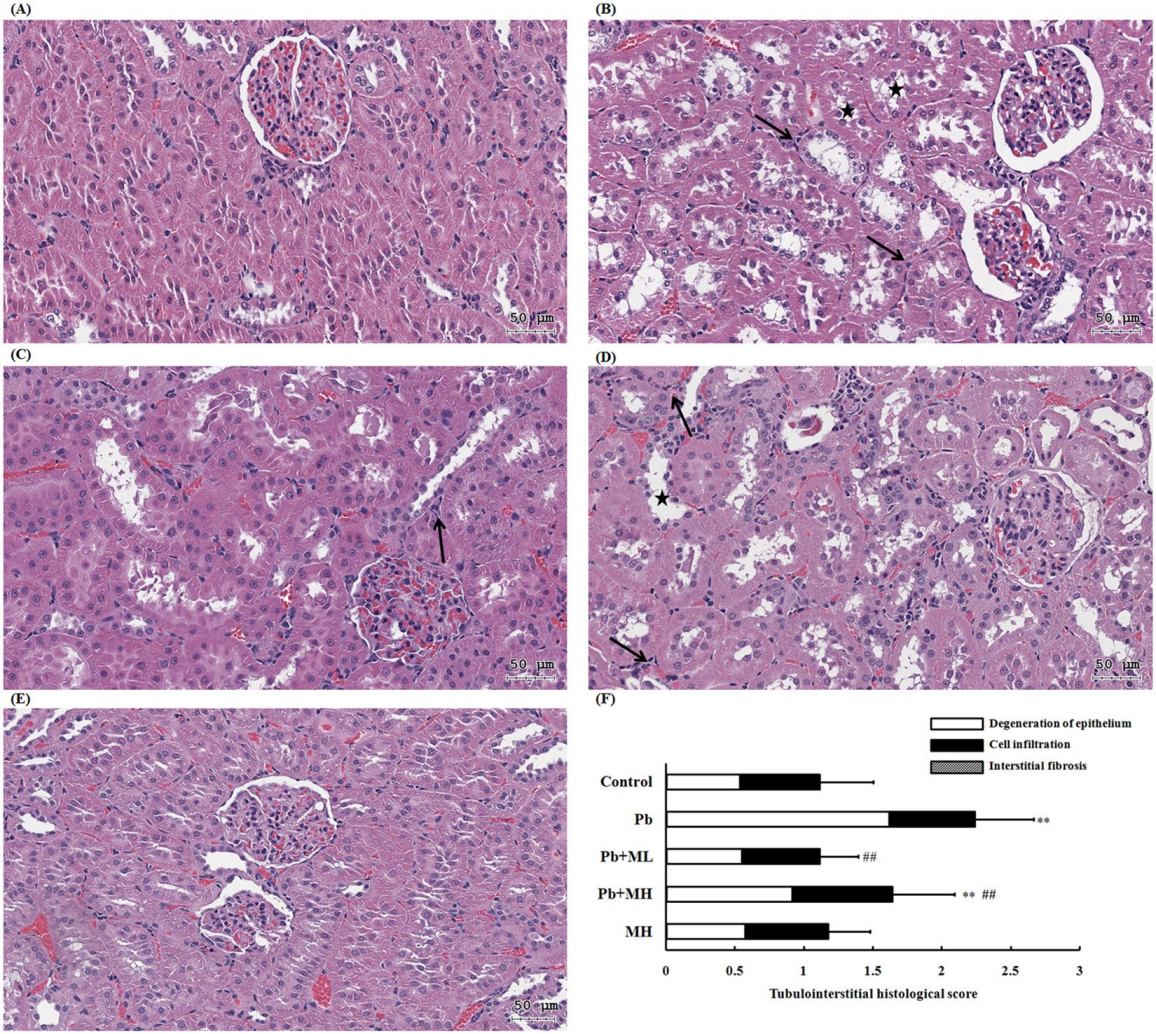
The kidney histological evaluation of the (**A**) control, (**B**) Pb, (**C**) Pb + ML, (**D**) Pb + MH, and (**E**) MH groups. (**F**) Sum of the tubulointerstitial histological score of Pb-induced nephrotoxicity rats that were co-treated with metformin or not. Scale bar, 50 μm. (★) means degeneration of tubular cell; (→) means inflammatory cell infiltration. **p < 0.01 compared with control group using one-way ANOVA; ^##^*p* < 0.01 compared with the Pb group using one-way ANOVA. In the control and MH groups, there was no obvious damage. In Pb-treated rats (Fig. 1B–D), the renal tissues displayed tubular degeneration and cell infiltration without interstitial fibrosis. Compared to the Pb + MH group, low-dose metformin (Pb + ML) showed more reno-protective ability.

In both the control and the MH group, no renal damage was seen (Fig. 2A,E). All other groups exhibited tubular apoptosis, proximal tubular karyomegaly, intranuclear inclusion body in the proximal tubule, and proximal tubular degeneration with inflammatory cells infiltration, but no glomerular damage. According to the sum of the tubulointerstitial histological score (Fig. 2F), Pb + ML attenuated renal damage compared with Pb rats. In addition, there was no obvious damage to liver tissues (Supplementary Fig. S1).

### Clinical chemistry

**Figure 3:**
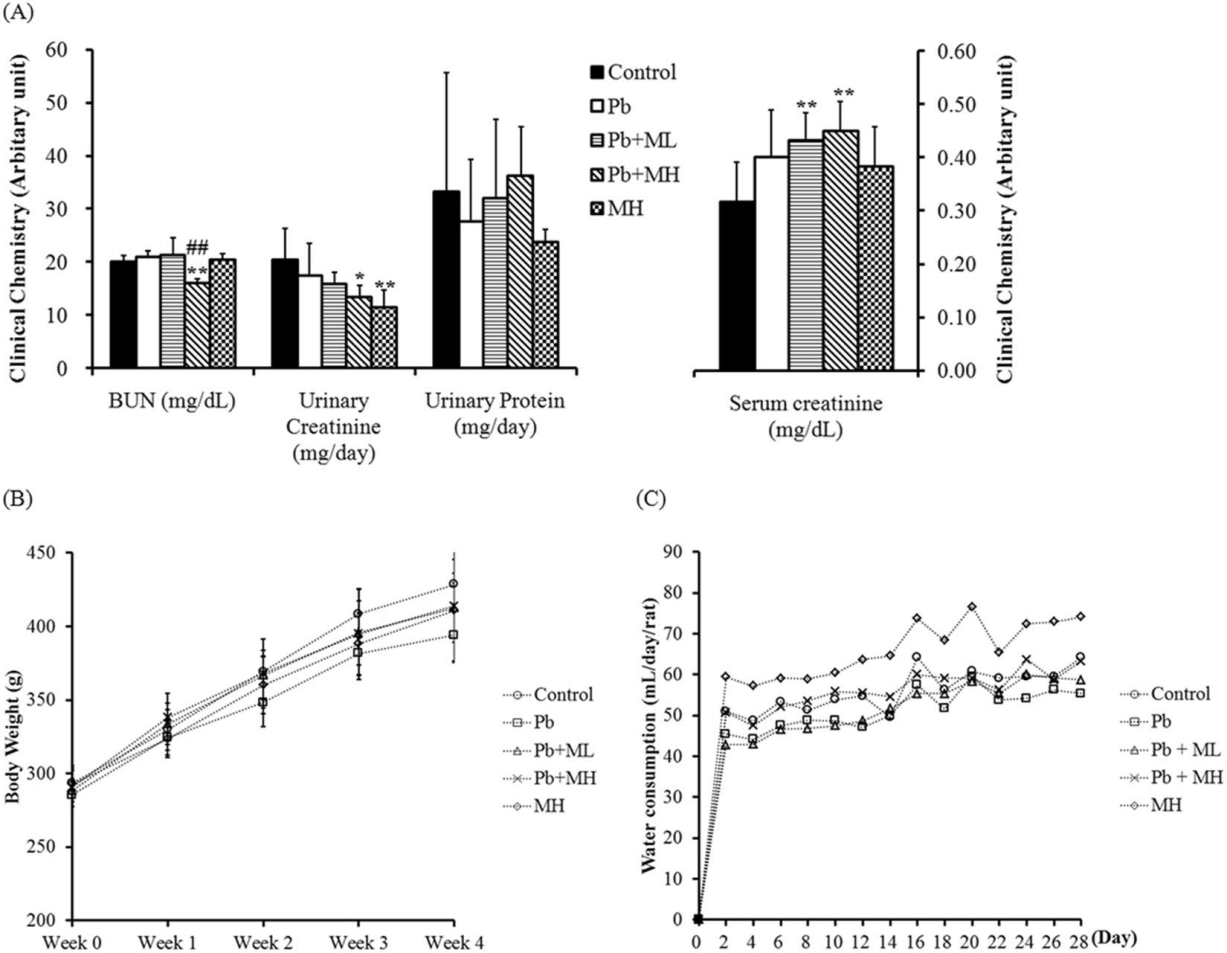
Effects of metformin on (**A**) clinical chemistry, (**B**) body weight and (**C**) water consumption in Pb-induced nephrotoxicity rats. *p < 0.05, **p < 0.01 compared with control group using one-way ANOVA; ^#^*p* < 0.05, ^##^*p* < 0.01 compared with Pb group using one-way ANOVA. In Pb-exposed rats, serum creatinine was marginally increased compared with control rats. After co-treatment with metformin, serum creatinine was increased in both Pb + ML and Pb + MH rats compared with control rats. BUN was reduced in Pb + MH rats compared with control and Pb rats. Pb-exposed rats had marginally decreased urinary creatinine compared with control rats. After co-treatment with metformin, urinary creatinine was significantly decreased in Pb + MH and MH rats compared with control rats. There were no significant differences in urinary protein, body weight or water consumption.

The Pb group had marginally increased serum creatinine (*p* = 0.052) and decreased urinary creatinine (*p* = 0.05) compared with the control (Fig. 3A). The Pb + ML group had significantly increased serum creatinine (*p* = 0.008) compared with the control group (Fig. 3A). The Pb + MH group had significantly increased serum creatinine (*p* = 0.003), and decreased BUN compared with the control (*p* = 0.001) and the Pb group (*p* < 0.000), as well as decreased urinary creatinine (*p* = 0.011) compared with the control group (Fig. 3A). In the MH group, urinary creatinine was significantly decreased compared with the control group (*p* = 0.002) (Fig. 3A). There were no significant differences in urinary protein, body weight or water consumption between groups (Fig. 3A–C). The serum AST/ALT ratio was not significantly different between groups (Supplementary Fig. S1).

### Metabolic profiling, multivariate analysis and identification of urine metabolites

**Figure 4:**
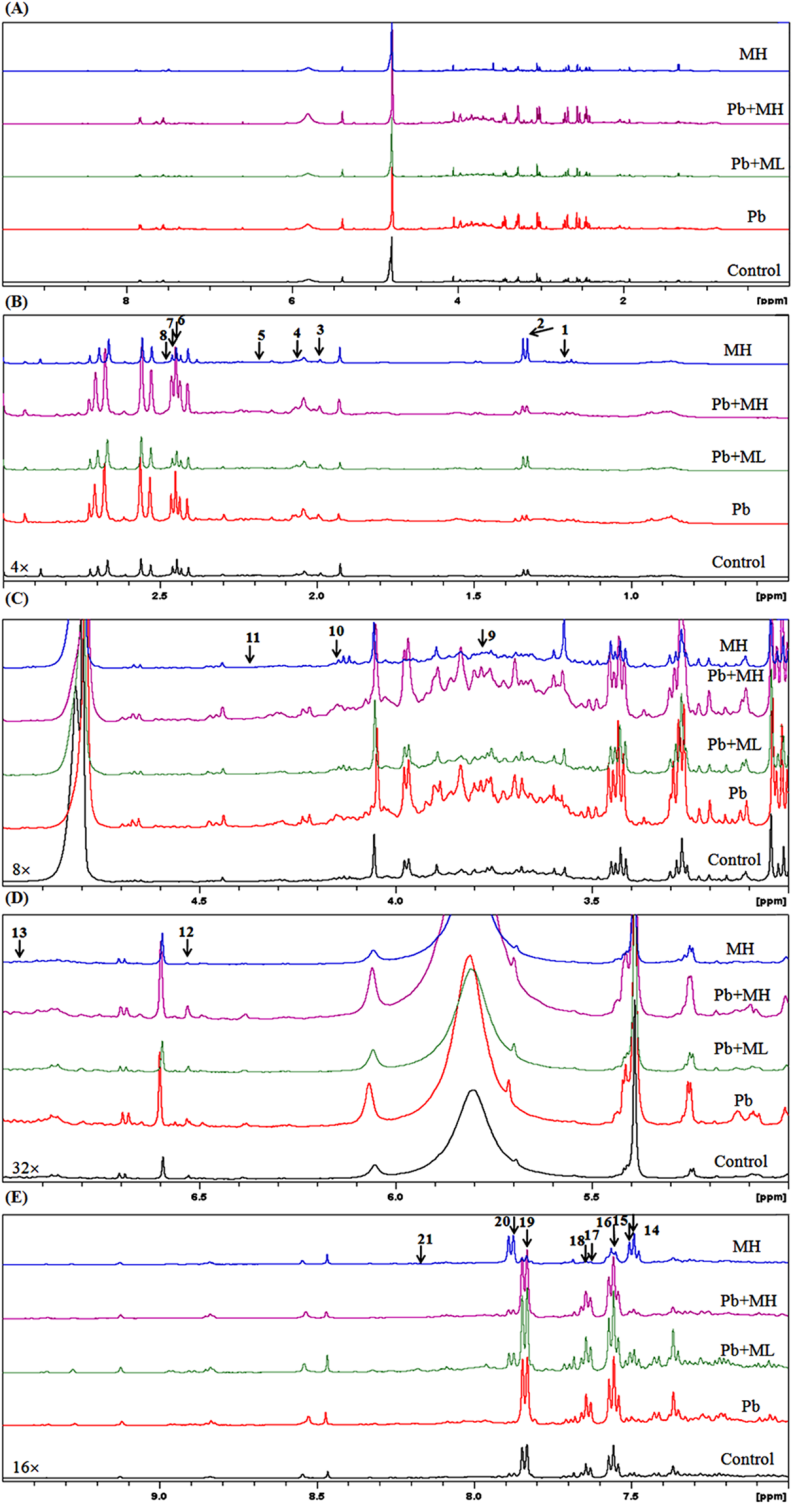
1D NMR spectrum of urinary metabolomics in Pb-induced nephrotoxicity rats co-treated with metformin. (**A**) Full spectra 0–9.5 ppm; (**B**) 0–3.5 ppm (4× magnification); (**C**) 3.5–5 ppm (8× magnification); (**D**) 5–7 ppm (32× magnification) and (**E**) 7–9.5 ppm (16× magnification). Arrows point to peaks that were significantly altered. There were 21 NMR peaks that were significantly altered between the 5 groups in 1D-NMR spectra according to one-way ANOVA.

**Figure 5:**
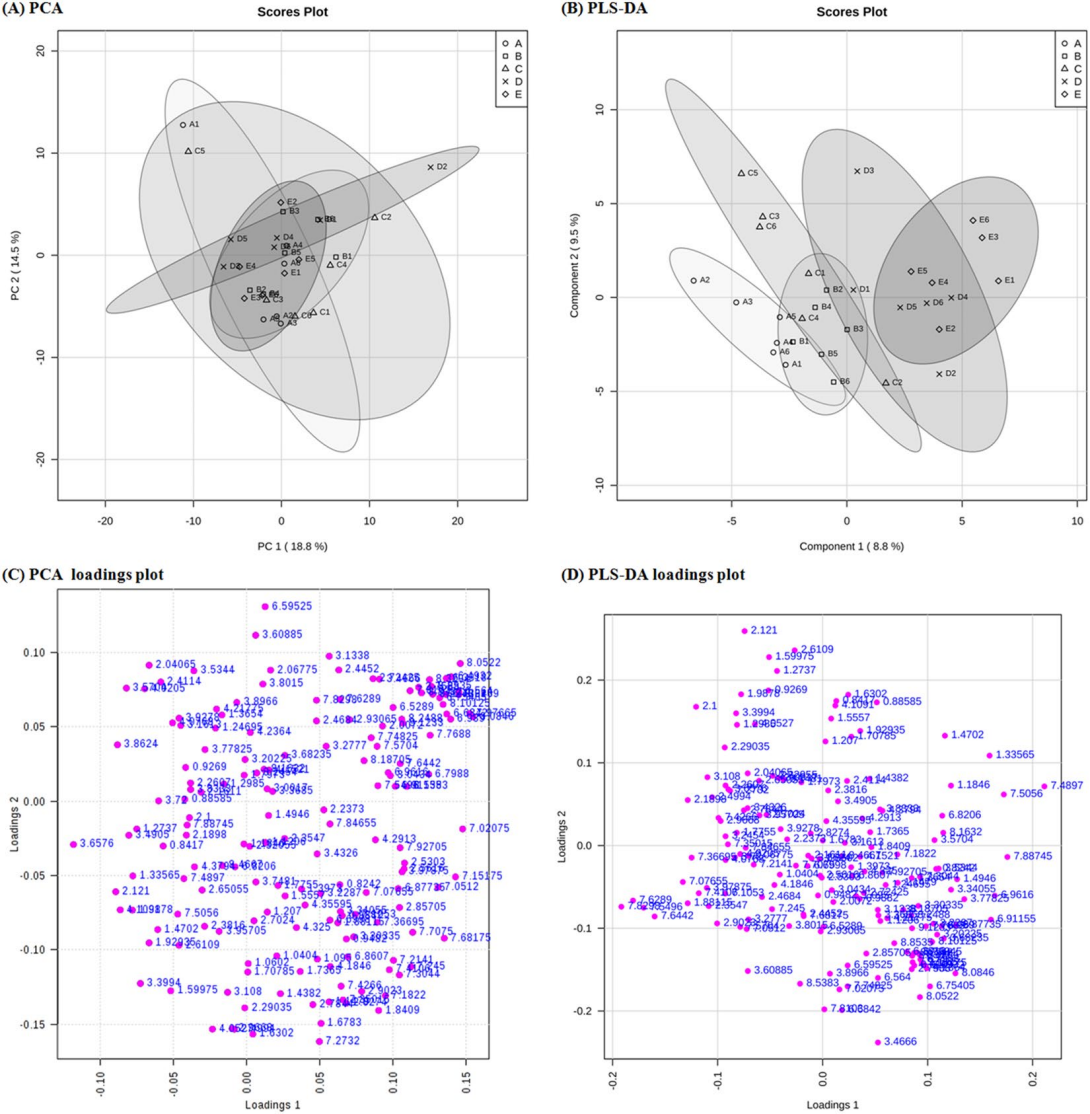
Multivariate analysis of (**A**) the PCA scores plot and (**B**) PLS-DA score plot. (**C**) PCA loading plot and (**D**) PLS-DA loading plot in urine samples of Pb-induced nephrotoxicity rats co-treated with metformin. (**A**–**E**) represents control, Pb, Pb + ML, Pb + MH and MH groups, respectively. In the PCA score plot, there was no obvious discrimination, and no obvious features were seen in the PCA loading plot. Therefore, PLS-DA was used to separate metabolites according to classification. The PLS-DA score plot shows good separation, and some features were obviously discriminated in the loading plot.

1D NMR spectra are shown in Fig. 4. Twenty-one peaks were significantly altered between the five groups (Fig. 4B–E; detailed in Supplementary Table S1). NMR spectra were integrated into peak lists followed by multivariate analysis. PCA analysis showed that there were no outliers. All samples were within the 0.95 Hotelling’s T2 ellipse in scores plot (Fig. 5A), and there was no obvious contribution in the loading plot (Fig. 5C). PLS-DA was further applied to analyse peaks according to classification. In PLS-DA analysis, there was good separation between the 5 groups (Fig. 5B), and there was some effective contribution in the loading plot (Fig. 5D). Leave-one-out cross validation (LOOCV) and the permutation test were used to validate the PLS-DA model. Since the difference between R^2^ and Q^2^ was greater than 0.3 in the LOOCV method (Supplementary Table S2), PLS-DA was successfully validated by the permutation test (*p* = 0.044). All significantly altered peaks were identified and are detailed in Supplementary Table S3. As a result, sixty-eight metabolites were identified, and fifty-four metabolites among them had a variable importance in projection (VIP) score greater than 1 (Supplementary Table S3).

To understand which pathways were involved in these metabolite changes, all significantly altered metabolites were submitted to pathway analysis. The crucial pathways were arginine and proline metabolism, d-glutamine and d-glutamate metabolism, and alanine, aspartate and glutamate metabolism (Supplementary Fig. S2). Based on our knowledge and interests, we chose seven potential biomarkers for further quantification: *N*-acetyl-β-d-glucosaminidase (NAG), 5-aminolevulinic acid (ALA), d- (d-LA) and l-lactate (l-LA), guanidinoacetic acid (GAA), indoleacetic acid (IAA), and hippuric acid (HA) in rat urine.

### Quantification of potential biomarkers

Quantitative data on these metaolites are shown in Table 1. Value of urinary d-LA and l-LA were well-established in our previous studies^13,27–29^. We validated ALA, GAA, IAA and HA, and all quantitative methods had a good linear range, and acceptable precision, accuracy and matrix effect (Supplementary Tables S4–6). Among these metabolites, urinary NAG and HA were not significantly altered. Urinary ALA (24.25 ± 6.55 vs. 0.55 ± 0.11, *p* = 0.0003), d-LA (0.68 ± 0.28 vs. 0.32 ± 0.13, *p* = 0.019) and GAA (26.31 ± 5.40 vs. 19.35 vs. 4.74, *p* = 0.039) were significantly increased in the Pb group compared with the control group. Low-dose metformin reduced urinary d-LA (0.32 ± 0.08 vs. 0.68 ± 0.28, *p* = 0.014), and high-dose metformin decreased urinary ALA (17.23 ± 7.43 vs. 24.25 ± 6.55, *p* = 0.044), compared with Pb rats. Finally, the MH group had increased urinary d-LA (0.99 ± 0.36 vs. 0.32 ± 0.13, *p* = 0.002), l-LA (9.44 ± 4.01 vs. 2.19 ± 1.00, *p = *0.001) and IAA (24.52 ± 4.80 vs. 14.51 ± 4.42, *p* = 0.001) compared with control rats.

**Table 1:** Metformin’s effects on potential biomarkers of Pb-induced nephrotoxicity in rat urine.

	NAG (U/L)	ALA (μg/mg Cr)	d-LA (μmol/mg Cr)	l-LA (μmol/mg Cr)	GAA (μg/mg Cr)	IAA (μg/mg Cr)	HA (mg/mg Cr)
Control	3.19 ± 1.00	0.55 ± 0.11	0.32 ± 0.13	2.19 ± 1.00	19.35 ± 4.74	14.51 ± 4.42	1.09 ± 0.28
Pb	2.39 ± 0.41	24.25 ± 6.55^**,$$^	0.68 ± 0.28^$^	4.03 ± 2.82	26.31 ± 5.40^$^	16.70 ± 6.00	1.30 ± 0.51
Pb + ML	2.93 ± 0.66	23.50 ± 8.10**	0.32 ± 0.08^@^	2.87 ± 0.62	33.21 ± 20.70	16.60 ± 3.97	1.40 ± 0.39
Pb + MH	2.68 ± 0.88	17.23 ± 7.43^**,#^	0.87 ± 0.59**	7.09 ± 4.95*	38.90 ± 15.07	21.30 ± 3.63	1.27 ± 1.08
MH	2.94 ± 0.76	0.94 ± 0.15	0.99 ± 0.36**	9.44 ± 4.01**	23.90 ± 8.58	24.52 ± 4.80**	0.68 ± 0.65

*N*-acetyl-β-d-glucosaminidase (NAG); 5-aminolevulinic acid (ALA); d-lactate (d-LA); l-lactate (l-LA); guanidinoacetic acid (GAA); indole-3-acetic acid (IAA); hippuric acid (HA) *p < 0.05, **p < 0.01 com*p*ared with control group using one-way ANOVA; ^#^*p* < 0.05 compared with Pb group using one-way ANOVA; ^$^*p* < 0.05, ^$$^*p* < 0.01 compared with control group using two-tailed Student’s t-test. ^@^*p* < 0.05 compared with Pb group using two-tailed Student’s t-test.

According to quantitative analysis, urinary NAG, IAA and HA were not significantly different between the 5 groups, except for urinary IAA in the MH group. In Pb-exposed rats, urinary ALA, d-LA and GAA were profoundly increased compared with control rats. After co-treatment with metformin, urinary ALA and d-LA were significantly decreased in Pb + MH and Pb + ML rats compared with Pb-exposed rats, respectively.

However, urinary GAA was not altered after co-treatment with metformin. In high-dose metformin (MH group), urinary d- and l-LA showed elevation compared with control rats.

## Discussion

To our knowledge, this is the first investigation of the effect of metformin on low-level Pb-induced renal injury in rats by urinary metabolomics. The routes of environmental exposure to Pb are inhalation, ingestion and dermal contact. After absorption, Pb particularly accumulates in the kidney and liver^30^. To inspect the lesion of Pb toxicity, both kidney and liver tissues were collected and evaluated by histopathology and clinical chemistry. Serum AST and ALT are not specific for the diagnosis of liver injury, and the AST/ALT ratio is more clinically useful^31^. Our results showed that 4-week oral administration of 250 ppm Pb did not influence liver tissues, as there was no elevation of the AST/ALT ratio and no liver damage. Although there was no obvious change in serum creatinine or BUN, renal tissues were certainly damaged by Pb (Fig. 2). Therefore, the following discussion focuses on Pb-induced nephrotoxicity.

Our study showed that Pb-induced renal injuries were related to nuclear inclusion bodies, proximal tubular apoptosis and inflammatory cell infiltration without glomerular damage. Our findings are consisted with other reports, which have shown that Pb binds to proteins and forms insoluble intranuclear inclusion bodies in proximal tubules^32^, and particularly damages proximal tubules^33^. In our clinical chemistry analysis, Pb did not alter serum creatinine or BUN compared with control. Even if serum creatinine and BUN are regarded as renal injury markers, these markers fail to increase in Pb-induced renal injury^11,12^. When these tests are found abnormal, the nephropathy has already reached the irreversible phase that may lead to renal insufficiency^14,34^. On the other hand, CKD increases in prevalence year by year across the world. Pb contamination is one of the causes of CKD, and it is not easy to diagnose^3^. Thus, the discovery of sensitive biomarkers for the early diagnosis of Pb-induced nephrotoxicity is urgent. After co-treatment with metformin, the alteration of creatinine in both serum and urine might be attributed to renal acidification^13^. Moreover, the reduction of BUN in the Pb + MH group might have been secondary to suppression of urea biosynthesis owing to inhibition of gluconeogenesis by high-dose metformin^35^. These data are consistent with our previous findings that reno-protection of metformin is more predominant in a low dose than a high dose^18,19^.

NMR-based metabolomics is a powerful tool for systematic investigation of potential biomarkers in biofluids^24^. Urine, a waste and filtrate from kidney, is non-invasive and directly reflects renal status^21,36^. Therefore, urinary metabolomics coupled with multivariate analysis provide a powerful approach to investigate metformin’s effect on Pb-induced renal injury. MetaboAnalyst, a comprehensive tool for metabolomics analysis and interpretation, provides an effective approach to discover the potential biomarkers of disease statuses^37^. In PCA analysis, there were no observations lying outside the 0.95 Hotelling’s T2 ellipse (Fig. 5A). The loading plot of PCA showed no significant contribution (Fig. 5C). Therefore, PLS-DA was used to discriminate according to classification. Since PLS-DA analysis can have a ‘data overfitting’ problem, LOOCV and the permutation test was used to validate our PLS-DA model^38^. In LOOCV, the difference between R^2^ and Q^2^ values was used as the indicator to determine if PLS-DA was overfitted. This difference should be less than 0.3^39^. In our study, the difference between R^2^ and Q^2^ was greater than 0.3 (Supplementary Table S2). Therefore, the permuation test was used to validate the PLS-DA model. The permutation test was performed in a 2,000-iteration re-analysis, and only 88 of those results were significantly better than the original dataset (*p* = 88/2000 = 0.044 < 0.05). Taking these data together, PLS-DA was validated and suitable for estimating potential biomarkers. According to the score plot of PLS-DA, the discrimination between the 5 groups was more effective in metformin-treatment than in Pb poisoning (Fig. 5B). The significant peaks were observed in the loading plot (Fig. 5D) and the VIP score was used to find the important biomarkers (detailed in Supplementary Table S3). Based on our interests and knowledge, we quantified 7 potential biomarkers (Table 1).

NAG is a lysosomal enzyme highly expressed in kidney. Though elevation of urinary NAG is positively correlated with renal injury^28^, it might fail to change in Pb-exposed workers, which is consistent with our study^40^. Altogether, urinary NAG might not be suitable for diagnosis of early low-level Pb-induced renal injury.

ALA, a precursor of haem synthesis, accumulates due to inhibition of ALA dehydratase (ALA-D) by Pb and is subsequently eliminated into urine^41^. The elevation of urinary ALA supports other studies that showed that it is highly increased after exposure to Pb^42^. However, some still hold the opposite opinion that urinary ALA is not a suitable indicator of exposure to Pb in human^43^. There is no direct correlation between renal morphology and urinary ALA level. To our knowledge, this is the first study that simultaneously determined urinary ALA and renal morphology in Pb-exposed rats. Our study showed that low-level Pb resulted in a 40-fold increase in urinary ALA compared with control rats. After co-treatment with a high dose of metformin, urinary ALA was profoundly decreased compared with Pb rats (Table 1). Even though metformin had no influence on ALAD activity *in vitro*^44^, the effect of metformin on ALAD activity remains unclear *in vivo*. On the other hand, metformin enhances ALA-related photodynamic therapy by activating AMPK autophagy signalling *in vitro*^45^; hence, the relationship between metformin and ALA still needs to be elucidated. According to our results, we regard urinary ALA as an early biomarker for diagnosis of low-level Pb-induced renal injury.

Lactate (LA), containing a chiral centre, has two enantiomers, d- and l-LA. d-LA, a downstream metabolite of methylglyoxal, is eliminated from kidney^46^. Due to the high reactivity and deleterious characteristics of methylglyoxal, accumulation of methylglyoxal and its metabolite, d-LA, is associated with renal injury^13,18,19,47^. l-LA is formed from pyruvate through l-lactate dehydrogenase^48^. In this study, urinary d-LA, not l-LA, was increased in Pb rats compared with control rats (Table 1). Similarly, our previous studies demonstrated that urinary d-LA is a powerful biomarker of renal injury in rodents^13,27,28^ and human^29^. After co-treatment with a low dose of metformin, urinary d-LA was reduced compared with Pb rats. In this regard, our previous study demonstrated that metformin could reduce renal methylglyoxal and urinary d-LA in Pb-induced renal injury rats^13^. Again, urinary d-LA was shown to be a promising biomarker for assessing renal injury. However, high-dose metformin did not attenuate Pb-induced renal injury, and it produced higher urinary d- and l-LA compared with control rats. High urinary l-LA might be attributed to inhibition of gluconeogenesis by high-dose metformin^49^. On the other hand, d-LA can be produced by intestinal *Lactobacillus* species^50^. Administration of metformin could change gut microbiota that enriches *Lactobacillus* in high-fat diet-induced obesity in rats^51^. We speculate that high urinary d-LA in high-dose metformin might be secondary to the alteration of gut microbiota. Taking these findings together, we suggest that low-dose metformin is more suitable for recovering renal injury in Pb-induced nephrotoxicity than high-dose.

GAA is an essential precursor of creatine and is synthesized by transamidation from glycine and arginine in the proximal tubules^52^. In nondialysed patients with chronic renal sufficiency, the reduction of creatinine clearance is concomitant with a decrease of GAA excretion^53^. The suppression of GAA excretion might be attributable to an inhibition of amidinotransferase or an impairment of renal production^54,55^. However, there is evidence that urinary GAA is initially increased and subsequently decreased in acute renal failure in rats^55^. Kuwagaki *et al*. demonstrated that the elevation of urinary GAA at day 1 is due to proximal tubular damage, which would cause less effective reabsorption of GAA. Then, inhibition of renal amidinotransferase activity leads to decreased urinary GAA^55^. Furthermore, urinary GAA is profoundly increased in traditional herbal medicine-induced nephrotoxicity^56^. Elevation of GAA excretion might have been secondary to disturbance of renal reabsorption in our study. This is the first study demonstrating that urinary GAA could be an early biomarker for low-level Pb-induced renal injury.

Recently, gut microbiota have received attention concerning renal injury^57^. Subchronic exposure to Pb interferes with intestinal microbiota such as *Ruminococcaceae*, *Akkermansia* and *Lachnoclostridium*^58,59^. In addition, metformin could significantly increase the abundances of gut microbiota, *Akkermansia muciniphila* and *Clostridium cocleatum*, in high-fat-diet induced obesity mice^60^. Hippuric acid or indoleacetic acid is the product of benzoate or tryptophan metabolism by a range of gut microbiota, for example *Clostridium* sp., *Bacteroides*, *Clostridia*, and *Escherichia coli*^61–64^. No reports revealed that urinary HA and IAA were related to Pb-induced nephrotoxicity with metformin treatment. However, both urinary HA and IAA were not significantly altered in low-level Pb exposure in this study (Table 1). Glomerular filtration contributes to HA excretion^65^. Therefore, the lack of alteration of urinary HA and IAA might be secondary to mild renal injury and might not influence the microbiota as a result of low-level Pb^58^. In the MH group, the elevation of IAA might attribute to the effect of metformin on gut microbiota mentioned above.

In human population, Lalau *et al*. investigated that patients were administered metformin 850 or 1,700 mg daily according to CKD stage 2 to 3 or 1 to 2, respectively. Authors concluded that metformin can be efficiently cleared in mild-to-moderate CKD patients^66^. In a population open cohort study showed that the risk of severe complications of diabetes was significantly decreased in metformin user compared to non-user^67^. A retrospective cohort study investigated the effect of initiators (metformin or sulfonylurea) on veterans with diabetes. The result showed that metformin treatment had a lower risk of decline in kidney function or death^68^. Inzucchi *et al*. proposed that a maximal daily dose of metformin in patients with CKD stage 1 to 3b are ranged from 2250 to 1000 mg daily^69^. In our study, 50 and 100 mg/kg of metformin in rats was equal to 606 and 1211 mg/day in human dosage, according to practice guide for dose conversion^70^. In present study, the low-dose metformin (50 mg/kg) provide the better renoprotective effect than the high-dose one. Based on our results, we speculated that administration of 600 mg metformin might possibly attenuate low-level Pb-induced renal injury in human population, especially in the early stage of renal injury without hyperglycemia.

**Figure 6:**
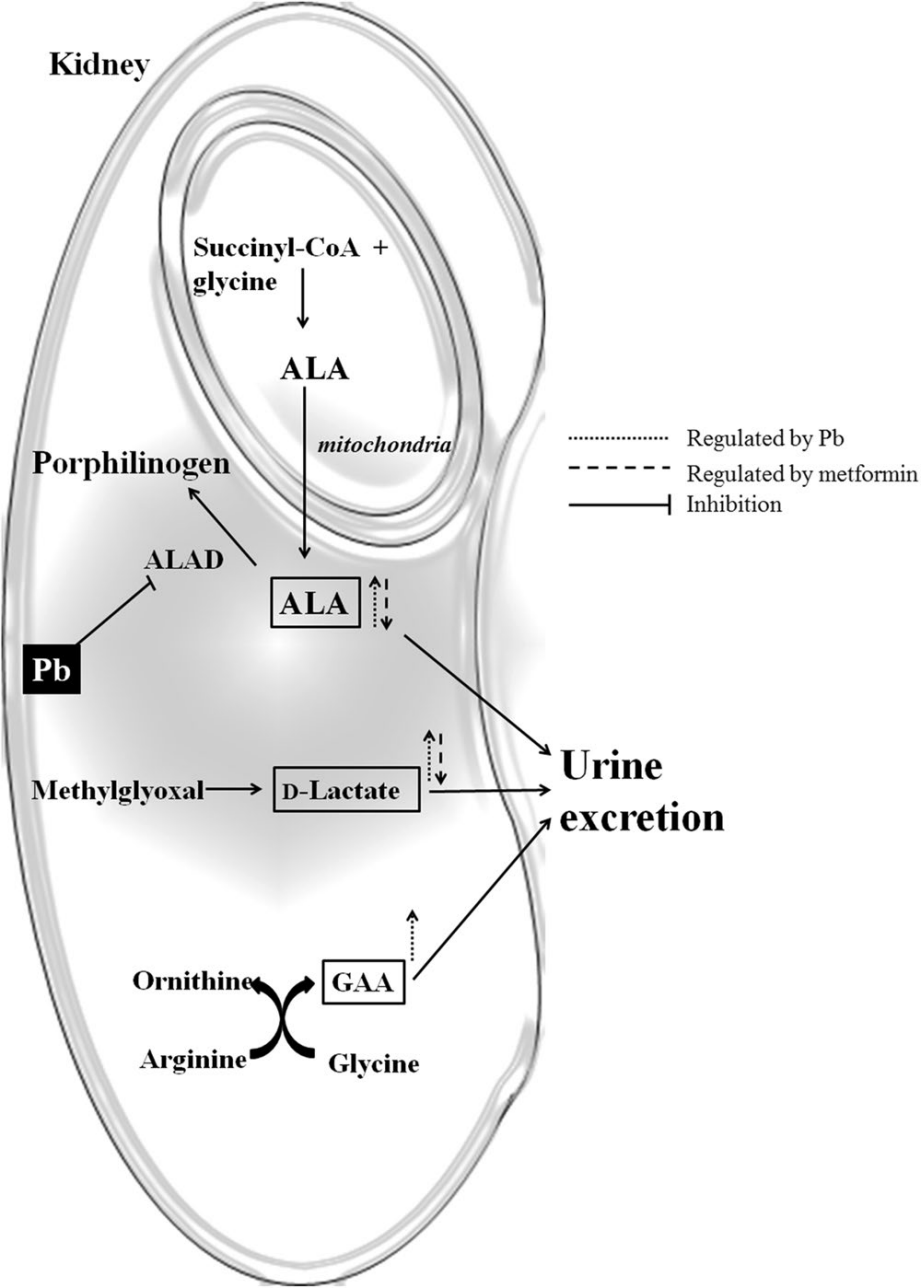
Hypothesis of metformin’s effect on low-level Pb-induced nephrotoxicity in rats. To our knowledge, this is the first study demonstrate that low-level Pb-induced renal injury is closely correlated with excretion of d-LA, ALA and GAA. The main protective mechanism of metformin is through the methylglyoxal pathway, which reduces d-LA excretion.

Altogether, the mechanism of low-level Pb-induced nephrotoxicity and reno-protection ability of metformin is described in Fig. 6. To our knowledge, this is the first study demonstrating that low-level Pb-induced renal injury is simultaneously related to excretion of d-LA, ALA and GAA. Although no new mechanisms of metformin were found, its reno-protection seemed to occur through reduced d-LA excretion through the methylglyoxal pathway. However, the possible mechanisms that metformin protects the kidney in rats might involve in activating AMP-activated protein kinase and antioxidant activities and suppressing interstitial fibrosis and inflammation responses^71–73^. But the assumption of renoprotective effects of metformin on Pb-induced nephrotoxicity still need to be confirmed by further studies. Additionally, high-dose metformin was more influential in metabolic profiling than low-dose, for example, lactate and IAA. These perturbances of metabolites in rats treated with high-dose metformin suggest that adequate doses of metformin have clinical utility.

## Conclusion

Combining metabolomics with quantitative analysis has promise as non-invasive tool to discover potential biomarkers. Our results suggest that the elevation of urinary d-LA, ALA and GAA can be biomarkers for early diagnosis of low-level Pb-induced nephrotoxicity. The reno-protective effect of metformin on Pb-induced nephrotoxicity might be attribuable to the reduction of d-LA excretion. Further studies should clarify the relationships between biomarkers and disease stage. The effects of different doses of metformin on metabolic pathways are worth studying.

## Materials and Methods

### Chemicals and reagents

Lead nitrate (Pb(NO_3_)_2_), sodium lauryl sulfate, creatinine anhydrous, propionic acid, lithium d-, and l-lactate, acetylacetone, 37% formaldehyde, cimetidine, indoleacetic acid, methylumbelliferone, and methylumbelliferyl *N*-acetyl-β-d-glucosaminide were purchased from Sigma-Aldrich Fine Chemicals Inc. (MO, USA). Formic acid, HPLC grade acetonitrile (ACN), ethanol and methanol (MeOH) were purchased from Merck (Darmstadt, Germany). Hippuric acid, 2,2′-dipyridyl disulfate (DPDS), triphenylphosphine (TPP) and 4-nitro-7-piperazino-2,1,3-benzoxadiazole (NBD-PZ) were purchased from Tokyo Chemical Industry Co., Ltd. (Tokyo, Japan). 5-Aminolevulinic acid hydrochloride and guanidinoacetic acid were purchased from Alfa Aesar (MA, USA). Sulbactam was purchased from Cayman Chemical (Michigan, USA). Acetic acid was purchased from Hayashi Pure Chemical Ind.,Ltd. (Osaka, Japan). Trifluoroacetic acid (TFA) was purchased from Riedel-de Haën (Seelze, Germany). 3-(Trimethylsilyl)propionic-2,2,3,3-d_4_ acid sodium salt (TSP) was purchased from Cambridge Isotope Laboratories (MA, USA). 1,1-Dimethylbiguanide hydrochloride (metformin) was purchased from Santa Cruz Biotechnology, Inc. (Texas, USA). Protein Assay Dye Reagent was purchased from Bio-Rad Laboratories (California, USA).

### Animal experiment, sample preparation and histopathology

Eight-week-old male Wistar rats (n = 30) were purchased from BioLASCO Taiwan Co., Ltd. (Taipei, Taiwan) and housed in the laboratory animal centre (Taipei Medical University). The animal experiment was conducted according to previous studies^13,33^. After 1 week of acclimation, rats were randomly divided into five groups: control (C), Pb only (Pb), Pb plus low dose of metformin (50 mg/kg/d) (Pb + ML), Pb plus high dose of metformin (100 mg/kg/d) (Pb + MH), and high dose of metformin only (MH). Pb (250 ppm) was given through drinking water, and metformin was given orally for 28 consecutive days. All animal experiments had been reviewed and approved by the Institutional Animal Care and Use Committee or Panel (Permit Number: LAC-2014-0286) to minimize pain and discomfort.

At the end of the experiment, twenty-four-hour urine was collected and centrifuged at 100 × *g* at 4 °C for 1 min. The supernatants were collected and stored at −80 °C. After that, rats were anaesthetized. Blood was collected and placed at room temperature at least 30 minutes for clotting, followed by centrifuging at 1200 × *g* for 10 minutes at 4 °C. Subsequently, sera were collected and stored at −80 °C.

Kidney and liver tissues were collected, immersed in 10% neutralized formalin and embedded in paraffin, followed by sectioning at 3 μm thickness. Haematoxylin and eosin (H&E) staining was used for investigating renal and hepatic morphology and was analysed in accordance with previous studies^13,74^.

### Clinical chemistry

Serum blood urea nitrogen (BUN), serum creatinine, alanine aminotransferase (ALT), aspartate aminotransferase (AST) were analysed by the Laboratory Animal Center of Taipei Medical University using VetTest^TM^ (IDEXX Laboratories, Inc., Westbrook, Maine, United States).

Determination of urinary creatinine was described in previous studies^13,27,28^. In brief, 20 μL of 2 mM cimetidine (as internal standard), 100 μL of urine and 400 μL of ACN were mixed together by vortexing for 1 minute, following by centrifuging at 875 × *g* for 15 minutes at 4 °C. The supernatants were collected and 10 μL was introduced into an HPLC-UV system at 234 nm wavelength. TSKgel ODS-80Ts QA (4.6 × 250 mm I.D., 5 μm) (TOSOH, Tokyo, Japan) was used. The mobile phase consisted of an aqueous phase (30 mM sodium lauryl sulfate and 100 mM potassium dihydrogen phosphate (adjusted to pH 3.0 using 85% o-phosphoric acid)) and an organic phase (ACN) (ratio of aqueous and organic phase = 12/7, v/v). The flow rate was set at 0.7 mL/min.

Urinary proteins were determined by the Bradford method^75^. In brief, 240 μL of urine was added to 60 μL of reagent dye and mixed well. Two hundred microliters of the mixture was transferred to a 96-well plate, and its absorbance was measured at 590 nm with a plate reader.

### 1D and 2D-NMR spectroscopy

The methods of NMR-based metabolomics from Xiao *et al*.^76^ and Zhang *et al*.^24^ were employed with minimal modification as described below^24,76^. Urine was further centrifuged at 9,700 × *g* for 10 minutes at 4 °C and the supernatant was collected. The supernatant (500 μL) was added to 50 μL of phosphate buffer in D_2_O (1.5 M potassium sodium phosphate buffer, pH 7.4), which contained 0.05% TSP (w/v) and 0.55% NaN_3_ (w/v), and the mixture was centrifuged at 9,700 × *g* for 10 min at 4 °C. The final supernatant (520 μL) was placed into a 5-mm NMR tube (Wilmad-Labglass, Vineland, NJ, USA) for 1D ^1^H-NMR, 2D ^1^H-^1^H TOCSY and ^1^H-^13^C HSQC. All NMR spectra were measured at 298 K on a 500 MHz Bruker Avance DRX 500 (Billerica, MA), employing a 5-mm inverse triple-resonance probe. Water suppression was executed using a standard NOESYGPPR1D pulse sequence (RD-G1-90°-t1-90°-tm-G2-90°-acq) with 16 ppm spectra width, 32 k data points, 128 transients and 1.5 s relaxation delay. 2D ^1^H-^1^H TOCSY was conducted with the ‘mlevgpph19’ pulse sequence with 2048 × 256 data points, 32 transients and 2.0 s relaxation time. ^1^H-^13^C HSQC was carried out with the ‘hsqcetgoorsiso2.2’ pulse sequence with 1024 × 128 data points, 128 transients and 1.5 s relaxation time.

### NMR data processing

NMR spectra were processed using Bruker TOPSPIN v3.2 (Bruker Biospin, Germany). The spectra were subjected to 1 Hz exponential broadening and zero-filled to 64 k points prior to Fourier transformation. Next, baseline and phase were manually corrected with the chemical shift referenced to TSP (δ 0.00 ppm). Due to an imperfect water signal and urea resonance, the chemical shifts between δ4.38 and 6.30 ppm were eliminated.

### Metabolite identification and pathway analysis

On the basis of multivariate analysis, the chemical shifts of 1D-NMR and 2D-NMR spectra were integrated into peak lists for the following assay. The lists of 1D-NMR were introduced into MetaboHunter for identification^77^. The shift tolerance was set at ±0.03 ppm. The lists of 2D-NMR spectra were introduced into MetaboMiner for identification^78^. The shift tolerance was set at ±0.03 ppm (for ^1^H) and ±0.1 ppm (for ^13^C). Metabolites were identified with the Human Metabolome Database and Madison Metabolomics Consortium Database. The metabolites that existed in urine were enrolled in our study for further analysis.

All significant metabolites were enrolled, and pathway analysis was carried out using Metaboanalyst 3.5. After uploading the identified compounds, those pathways with impact value >0.2 and *p* value < 0.05 were regarded as the most important pathways.

### Quantitative approach for estimating the potential biomarkers in urine

#### *N*-Acetyl-β-d-glucosaminidase (NAG)

Urinary NAG was measured as described in previous study^28^. In brief, 25 μL of urine was added to 20 μL of 4-methylumbelliferyl *N*-acetyl-β-d-glucosaminide (2.25 mM) and 80 μL of citrate buffer (100 mM, pH 4.6–5) at 37 °C for 15 minutes. Then, 100 μL of glycine buffer (200 mM, pH 10.4–10.65) was added to stop reaction at room temperature. The resultants (200 μL) were transferred into a 96-well plate and measured at 370/460 nm (excitation/emission wavelength) with a plate reader.

#### 5-Aminolevulinic acid (ALA)

Urinary ALA was determined according to a previously published method^79^. In brief, 10 μL of urine with 90 μL of 10% formaldehyde, and 700 μL of reagent (contained acetylacetone, ethanol and ddH_2_O = 1.5: 1: 7.5, v/v/v) were mixed and placed into boiling water for 15 minutes, then cooled down on ice. Then, 20 μL of ALA-derivatives were separated on a TSK-GEL ODS-Ts (4.6 × 150 mm I.D., 5 μm) with HPLC with a fluorescence detector at 370 nm excitation and 460 nm emission wavelength. The column oven and flow rate were set at 40 °C and 0.7 mL/min, respectively. The mobile phase was composed of methanol:water:acetic acid (50:50:1, v/v/v).

#### d- and l-Lactate (d- and l-LA)

d-LA and l-LA were measured in our laboratory using a well-established column-switching system^13,27–29,80^. In brief, urine (20 μL) and internal standard (10 μL, 1 mM propionic acid) were mixed with ACN (170 μL), and the mixture was centrifuged at 1000 × *g* for 10 minutes at 4 °C. The supernatants (100 μL) were derivatized with 100 μL of 8 mM NBD-PZ, and 50 μL of 280 mM DPDS and TPP at 30 °C for 3 hours. After that, 0.1% trifluoroacetic acid_(aq)_ (100 μL) was added to terminate the reaction. The resultant solution was eluted through the MonoSpin^TM^ SCX cartridge (GL Science Inc., Tokyo, Japan) eliminate excesses derivatization reagents^81^. The eluent (20 μL) was injected into the column-switching system to quantify d- and l-lactate in urine. The 1^st^ dimension of total lactate was separated on an ODS column (250 mm × 4.6 mm ID; 5 μm particle size; Biosil Chemical Co. Ltd., Taipei, Taiwan). The mobile phase was composed of 12% ACN, 20% MeOH and 68% ddH_2_O (v/v/v). The flow rate was set at 0.7 mL/min, and the oven was set at 30 °C. The 2^nd^ dimension of chiral separation was performed on a Chiralpak AD-RH column (150 mm × 4.6 mm ID; 5 μm particle size; Daicel Co., Osaka, Japan) at room temperature. The mobile phase was composed of 60% ACN and 40% ddH_2_O (v/v) and the flow rate was 0.3 mL/min. The 1^st^ and 2^nd^ dimensions were detected at an excitation and emission wavelength of 491 and 547 nm, respectively.

#### Guanidinoacetic acid (GAA), hippuric acid (HA) and indoleacetic acid (IAA)

Urinary GAA, HA and IAA were quantified with an Agilent 6470 triple quadrupole coupled with an Agilent 1260 Infinity II Quaternary Pump LC system (Waldbronn, Germany) according to a published article with some modifications^56^. In brief, 40 μL of urine was mixed with 10 μL of sulbactam (SB) (5 μg/mL, internal standard) and 160 μL ACN for deproteinization. After mixing for 3 minutes, the mixture was centrifuged at 16,400 × *g* for 8 minutes. The supernatant (1 μL) was introduced to liquid chromatography coupled with tandem-mass spectrometry (LC-MS/MS) and separated on a CAPCELL PAK C18 MGII (2.0 × 150 mm I.D., 5 μm) column. The mobile phase consisted of (A) water with 0.1% formic acid and (B) ACN with 0.1% formic acid, and the flow rate was 0.3 mL/min. The gradient was as follows: 0–2.5 min 80% A, 2.6–8 min 30% A, 8.1–14 min 0% A (for washing column), and 14.1–20 min 80% A (for column equilibration). The column oven was set at 30 °C. For multiple reaction monitoring (MRM), a quantifier and a qualifier were used to determine GAA, HA and IAA as shown in Table 2. Chromatogram of these metabolites was showed in Supplementary Fig. S3.

**Table 2:** Multiple reaction monitoring (MRM) parameters of GAA, HA and IAA.

Parameters	GAA	HA	IAA	SB
Precursor Ion **(**m/z**)**	118.1	180.1	176.1	232
Fragmentor **(**V**)**	90	60	91	90
Quantifier transition production **(**m/z**)** with CE **(**V**)**	76 (12)	105 (12)	130.1 (16)	187.9 (10)
Qualifier transition production **(**m/z**)** with CE **(**V**)**	101 (8)	77 (36)	103.1 (36)	140 (10)
CAV **(**V**)**	4	4	4	4
Dwell time **(**ms**)**	30	30	30	30
Polarity	(+)	(+)	(+)	(−)

Guanidinoacetic acid (GAA); hippuric acid (HA); indoleacetic acid (IAA); sulbactam (SB); cell accelerator voltage (CAV); collision energy (CE).

For mass spectrometry, dry gas temperature, dry gas flow, nebulizer, sheath gas temperature, sheath gas flow, capillary voltage, EMV voltage and nozzle voltage were set at 300 °C, 5 L/min, 35 psi, 250 °C, 11 L/min, 4000 V for positive and −3500 V for negative mode, 250 V (both positive and negative mode) and 500 V, respectively.

#### Multivariate and univariate statistical analysis

NMR spectra were integrated into peak lists and then introduced into the Metaboanalyst 3.5 website for multivariate analysis^82^. Data processing was according to user guideline of Metaboanalyst 3.5, and all data was normalized by sum of the spectrum followed auto-scaling.

Principal component analysis (PCA) was performed to examine the intrinsic outliers of the data set. The PCA score plot was used to reveal observations lying outside the 0.95 Hotelling’s T2 ellipse (strong outliers), and the loading plot is used to explain the pattern of score plot.

Partial least squares-discriminant analysis (PLS-DA) was used to find the best discriminant function model. The PLS-DA loading plot described the contribution of metabolites. However, PLS-DA analysis can be marred by a ‘data overfitting’ problem. Leave-one-out-cross validation (LOOCV) and the permutation test (n = 2000) were used to validate the PLS-DA model. In LOOCV, Q^2^ and R^2^ mean the robustness and reliance of the PLS-DA model, respectively. The difference between R^2^ and Q^2^ values was used as the indicator to determine if PLS-DA was overfitted, and it ideally is less than 0.3^39^. If not, permutation test should be used to validate the multivariate analysis model. The VIP score is used to estimate the important metabolites in a PLS-DA model. Among the identified metabolites, both VIP score (>1) and *p*-value (<0.05) were used to identify the potential biomarkers.

All quantitative data are expressed as the mean ± SD. They were compared using one-way ANOVA with the post hoc LSD test, or the two-tailed Students’ t-test. If the *p* value was less than 0.05, it represented a significant difference.

## Electronic supplementary material

Supplementary Information
