# Supplementary material for: Metabolic profiling of metformin treatment for low-level Pb-induced nephrotoxicity in rat urine
Source: Sci Rep. 2018 Oct 1;8:14587. doi: 10.1038/s41598-018-32501-3 (PMC6167321; doi:10.1038/s41598-018-32501-3)
Supplement: Supplementary file 1 — Supplementary Information [file 41598_2018_32501_MOESM1_ESM.docx]

Metabolic profiling of metformin treatment for low-level Pb-induced nephrotoxicity in rat urine

Yu-Shen Huang^1^, Shwu-Huey Wang^2,3^, Shih-Ming Chen^1,*^& Jen-Ai Lee^1,*^

^1^School of Pharmacy, College of Pharmacy, Taipei Medical University, 250 Wuxing St., Taipei, Taiwan

^2^Core Facility Center, Department of Research Development, Taipei Medical University, 250 Wuxing St., Taipei, Taiwan

^3^Department of Biochemistry and Molecular Cell Biology, School of Medicine, Taipei Medical University, 250 Wuxing St., Taipei, Taiwan


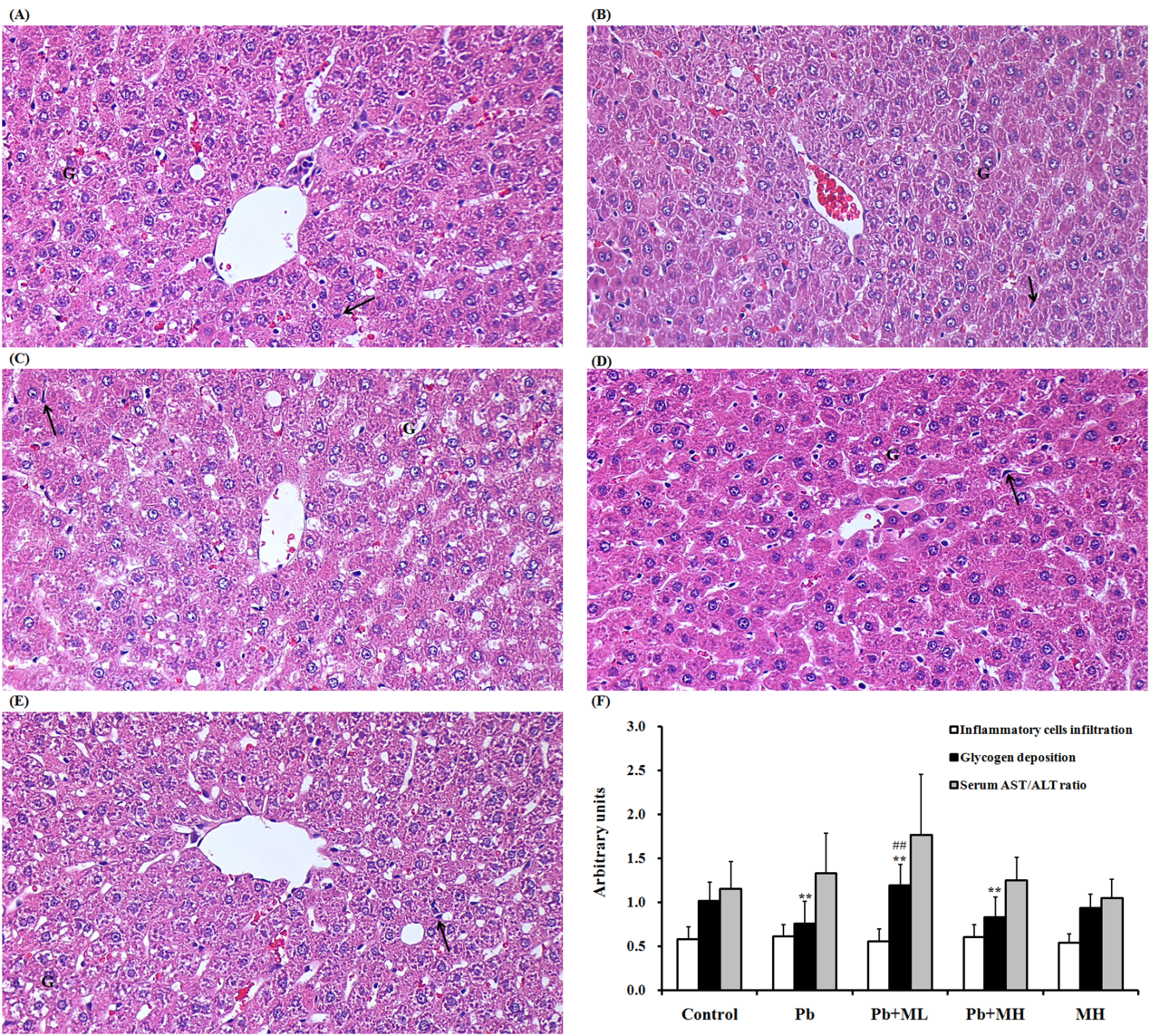


Supplementary Fig. S1. The liver histological evaluation and clinical chemistry of the (A) control, (B) Pb, (C) Pb+ML, (D) Pb+MH, and (E) MH groups. (F) The hepatic histological score and serum AST/ALT ratio of Pb-induced toxicity rats that were co-treated with metformin or not. 200× magnification.

(G) means glycogen deposition; (→) means inflammatory cells infiltration.

^**^*p* < 0.01 compared with the control group using one-way ANOVA; ^##^*p* < 0.01 compared with the Pb group using one-way ANOVA.

In hepatic tissues, there were simultaneous observation both cell infiltration and glycogen deposition between the 5 groups. Serum AST/ALT ratio were no significant difference. Therefore, Pb-induced toxicity was no effect on liver tissues.


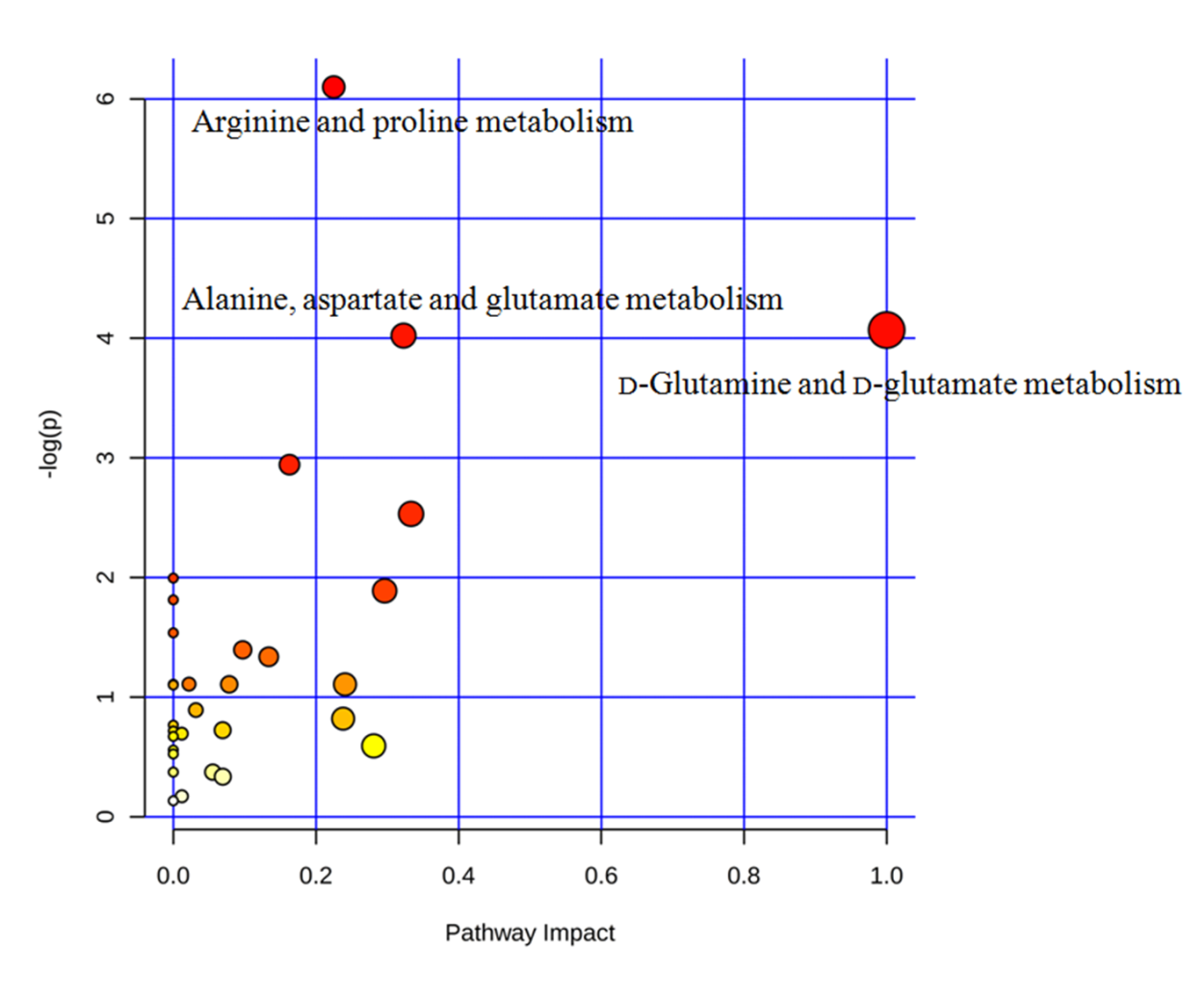


Supplementary Fig. S2. Pathway analysis through Metaboanalyst 3.5.

According to pathway analysis, pathway impact > 0.2 and *p*-value < 0.05 identified the important pathways. Amino acid metabolism pathways were the most important in our study, for example, arginine and proline metabolism, alanine, aspartate and glutamate metabolism, and d-glutamine and d-glutamate metabolism.


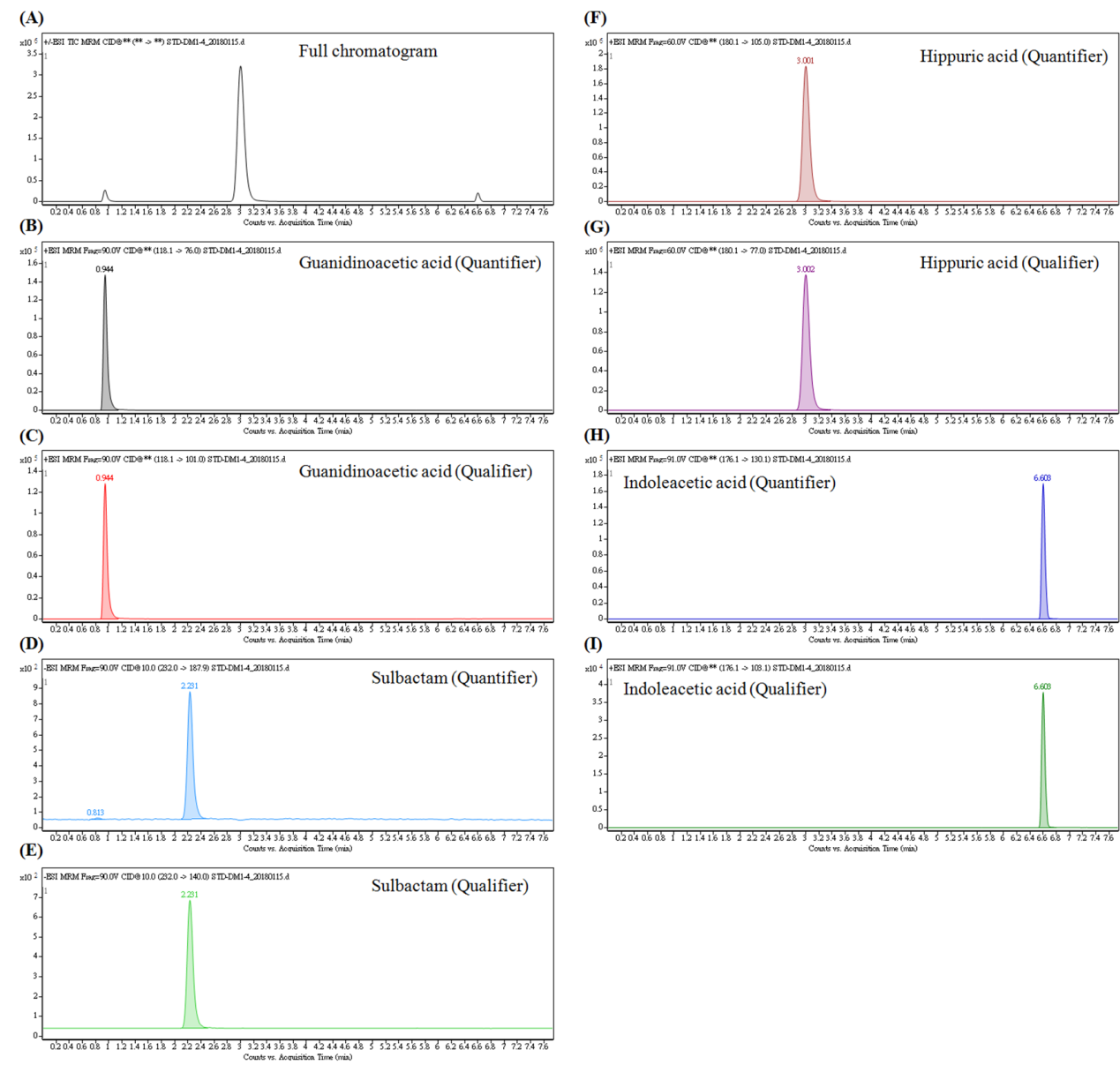


Supplementary Fig. S3. LC-MS/MS chromatogram of (A) full spectra, (B) quantifier of gaunidinoacetic acid, (C) qualifier of guanidinoacetic acid, (D) quantifier of sulbactam, (E) qualifier of sulbactam, (F) quantifier of hippuric acid, (G) qualifier of hippuric acid, (H) quantifier of indoleacetic acid, and (I) qualifier of indoleacetic acid.

Supplementary Table S1. The lists of significant altered peaks using univariate and multivariate analysis

| No. | Chemical shift (ppm, δ) | *p* value | -log10(p) | FDR | VIP score |
| --- | --- | --- | --- | --- | --- |
| 1 | 1.207 | 0.039 | 1.413 | 0.378 | 0.930 |
| 2 | 1.336 | 0.020 | 1.690 | 0.341 | 1.725 |
| 3 | 1.988 | 0.026 | 1.577 | 0.358 | 0.800 |
| 4 | 2.068 | 0.011 | 1.960 | 0.296 | 0.754 |
| 5 | 2.190 | 0.012 | 1.935 | 0.296 | 1.584 |
| 6 | 2.445 | 0.044 | 1.352 | 0.378 | 0.337 |
| 7 | 2.468 | 0.037 | 1.428 | 0.378 | 0.920 |
| 8 | 2.499 | 0.028 | 1.554 | 0.358 | 1.320 |
| 9 | 3.778 | 0.047 | 1.325 | 0.378 | 1.413 |
| 10 | 4.152 | 0.033 | 1.487 | 0.370 | 0.324 |
| 11 | 4.379 | 0.015 | 1.827 | 0.296 | 1.270 |
| 12 | 6.529 | 0.006 | 2.224 | 0.249 | 0.080 |
| 13 | 6.962 | 0.045 | 1.350 | 0.378 | 2.113 |
| 14 | 7.490 | 0.000 | 3.369 | 0.071 | 2.315 |
| 15 | 7.506 | 0.015 | 1.838 | 0.296 | 1.704 |
| 16 | 7.550 | 0.033 | 1.478 | 0.370 | 1.853 |
| 17 | 7.629 | 0.005 | 2.314 | 0.249 | 1.928 |
| 18 | 7.644 | 0.016 | 1.797 | 0.296 | 1.731 |
| 19 | 7.830 | 0.002 | 2.789 | 0.136 | 2.151 |
| 20 | 7.887 | 0.025 | 1.600 | 0.358 | 1.627 |
| 21 | 8.163 | 0.048 | 1.323 | 0.378 | 1.399 |

False discovery rate (FDR); variable importance in projection (VIP)

Supplementary Table S2. The results of leave-one-out-cross-validation (LOOCV) method in the PLS-DA model.

| Components | 1 | 2 | 3 | 4 | 5 |
| --- | --- | --- | --- | --- | --- |
| R^2^ | 0.7010 | 0.8588 | 0.9284 | 0.9696 | 0.9823 |
| Q^2^ | 0.1636 | 0.1933 | 0.0649 | 0.0182 | 0.0397 |

Supplementary Table S3. The lists of metabolites were identified through 1D and/or 2D NMR spectra.

| No. | Metabolite ID | HMDB | MMCD | HSQC | TOCSY | *p* value | FDR | VIP score |
| --- | --- | --- | --- | --- | --- | --- | --- | --- |
| 1 | (S)-3-Hydroxybutyric acid | **√** |  |  |  | 0.039 | 0.378 | 0.930 |
| 2 | *N*-Acetylornithine |  | **√** |  |  | 0.033 | 0.370 | 0.324 |
| 3 | 1-Methylhistidine |  | **√** | **√** |  | 0.016 | 0.296 | 1.731^*^ |
| 4 | 3-Hydroxymethylglutaric acid | **√** |  |  |  | 0.037 | 0.378 | 0.920 |
| 5 | 4-Acetamidobutanoic acid |  | **√** |  |  | 0.012 | 0.296 | 1.584^*^ |
| 6 | 4-Hydroxycinnamic acid |  | **√** |  |  | 0.000 | 0.071 | 2.315^*^ |
|  |  |  |  |  |  | 0.015 | 0.296 | 1.704^*^ |
| 7 | 5-Aminolevulinic acid^a^ | **√** |  |  |  | 0.028 | 0.358 | 1.320^*^ |
| 8 | Acetoin |  | **√** |  |  | 0.020 | 0.341 | 1.725^*^ |
|  |  |  |  |  |  | 0.012 | 0.296 | 1.584^*^ |
| 9 | Acetylcysteine |  | **√** |  |  | 0.015 | 0.296 | 1.270^*^ |
| 10 | Alpha-Hydroxyisobutyric acid | **√** |  |  |  | 0.020 | 0.341 | 1.725^*^ |
| 11 | Benzoic acid |  | **√** |  |  | 0.000 | 0.071 | 2.315^*^ |
| 12 | Caproic acid | **√** |  |  |  | 0.020 | 0.341 | 1.725^*^ |
| 13 | Cholic acid | **√** |  |  |  | 0.011 | 0.296 | 0.754 |
| 14 | Citramalic acid | **√** |  |  |  | 0.020 | 0.341 | 1.725^*^ |
| 15 | Citric acid |  | **√** |  | **√** | 0.028 | 0.358 | 1.320^*^ |
| 16 | Cotinine | **√** |  |  |  | 0.015 | 0.296 | 1.704^*^ |

Supplementary Table S3. The lists of metabolites were identified through 1D and/or 2D NMR spectra (continue).

| No. | Metabolite ID | HMDB | MMCD | HSQC | TOCSY | *p* value | FDR | VIP score |
| --- | --- | --- | --- | --- | --- | --- | --- | --- |
| 17 | Cyanocobalamin | **√** |  |  |  | 0.012 | 0.296 | 1.584^*^ |
|  |  |  |  |  |  | 0.028 | 0.358 | 1.320^*^ |
|  |  |  |  |  |  | 0.044 | 0.378 | 0.337 |
| 18 | Decanoylcarnitine | **√** |  |  |  | 0.037 | 0.378 | 0.920 |
| 19 | Dimethylamine | **√** |  | **√** |  | 0.028 | 0.358 | 1.320^*^ |
| 20 | d-Lactic acid**^a^** | **√** | **√** |  | **√** | 0.020 | 0.341 | 1.725^*^ |
| 21 | d-Xylitol |  | **√** |  |  | 0.047 | 0.378 | 1.413^*^ |
| 22 | Erythritol |  | **√** |  |  | 0.047 | 0.378 | 1.413^*^ |
| 23 | Folic acid | **√** | **√** |  |  | 0.000 | 0.071 | 2.315^*^ |
|  |  |  |  |  |  | 0.015 | 0.296 | 1.704^*^ |
|  |  |  |  |  |  | 0.012 | 0.296 | 1.584^*^ |
|  |  |  |  |  |  | 0.011 | 0.296 | 0.754 |
| 24 | Gluconic acid |  | **√** |  |  | 0.047 | 0.378 | 1.413^*^ |
| 25 | Glycerol |  | **√** |  | **√** | 0.047 | 0.378 | 1.413^*^ |
| 26 | Glycylproline | **√** |  |  |  | 0.026 | 0.358 | 0.800 |
| 27 | Guaiacol |  | **√** |  |  | 0.045 | 0.378 | 2.113^*^ |
| 28 | Guanidoacetic acid**^a^** | **√** | **√** |  |  | 0.047 | 0.378 | 1.413^*^ |

Supplementary Table S3. The lists of metabolites were identified through 1D and/or 2D NMR spectra (continue).

| No. | Metabolite ID | HMDB | MMCD | HSQC | TOCSY | *p* value | FDR | VIP score |
| --- | --- | --- | --- | --- | --- | --- | --- | --- |
| 29 | Hippuric acid**^a^** |  | **√** |  | **√** | 0.005 | 0.249 | 1.928^*^ |
|  |  |  |  |  |  | 0.033 | 0.370 | 1.853^*^ |
|  |  |  |  |  |  | 0.015 | 0.296 | 1.704^*^ |
| 30 | Homocysteine |  | **√** |  |  | 0.012 | 0.296 | 1.584^*^ |
|  |  |  |  |  |  | 0.011 | 0.296 | 0.754 |
| 31 | Hydrocinnamic acid | **√** |  |  |  | 0.028 | 0.358 | 1.320^*^ |
|  |  |  |  |  |  | 0.037 | 0.378 | 0.920 |
| 32 | Hydroxyproline | **√** | **√** |  |  | 0.044 | 0.378 | 0.337 |
| 33 | Indole | **√** | **√** |  | **√** | 0.033 | 0.370 | 1.853^*^ |
|  |  |  |  |  |  | 0.016 | 0.296 | 1.731^*^ |
| 34 | Indoleacetic acid**^a^** | **√** | **√** |  |  | 0.000 | 0.071 | 2.315^*^ |
|  |  |  |  |  |  | 0.005 | 0.249 | 1.928^*^ |
|  |  |  |  |  |  | 0.016 | 0.296 | 1.731^*^ |
|  |  |  |  |  |  | 0.015 | 0.296 | 1.704^*^ |
| 35 | Indolelactic acid | **√** | **√** |  |  | 0.000 | 0.071 | 2.315^*^ |
|  |  |  |  |  |  | 0.015 | 0.296 | 1.704^*^ |
| 36 | Indoxyl sulfate | **√** |  |  |  | 0.015 | 0.296 | 1.704^*^ |
| 37 | Isocitric acid | **√** |  |  |  | 0.028 | 0.358 | 1.320^*^ |
| 38 | Ketoleucine |  | **√** |  |  | 0.011 | 0.296 | 0.754 |

Supplementary Table S3. The lists of metabolites were identified through 1D and/or 2D NMR spectra (continue).

| No. | Metabolite ID | HMDB | MMCD | HSQC | TOCSY | *p* value | FDR | VIP score |
| --- | --- | --- | --- | --- | --- | --- | --- | --- |
| 39 | l-Acetylcarnitine |  | **√** | **√** |  | 0.028 | 0.358 | 1.320^*^ |
| 40 | l-Alanine |  | **√** | **√** | **√** | 0.047 | 0.378 | 1.413^*^ |
| 41 | l-Fucose | **√** |  |  |  | 0.047 | 0.378 | 1.413^*^ |
|  |  |  |  |  |  | 0.039 | 0.378 | 0.930 |
| 42 | l-Glutamic acid |  | **√** | **√** |  | 0.011 | 0.296 | 0.754 |
| 43 | l-Isoleucine |  | **√** | **√** | **√** | 0.039 | 0.378 | 0.930 |
| 44 | l-Kynurenine |  | **√** |  |  | 0.033 | 0.370 | 0.324 |
| 45 | l-Lactic acid^a^ | **√** |  | **√** |  | 0.020 | 0.341 | 1.725^*^ |
| 46 | l-Norleucine |  | **√** |  |  | 0.020 | 0.341 | 1.725^*^ |
| 47 | l-Proline |  | **√** |  | **√** | 0.026 | 0.358 | 0.800 |
|  |  |  |  |  |  | 0.011 | 0.296 | 0.754 |
| 48 | Mannitol | **√** |  |  | **√** | 0.047 | 0.378 | 1.413^*^ |
| 49 | Methylsuccinic acid | **√** |  | **√** |  | 0.028 | 0.358 | 1.320^*^ |
| 50 | Monomethyl glutaric acid | **√** |  |  |  | 0.012 | 0.296 | 1.584^*^ |
| 51 | *N*-Acetyl-d-glucosamine^a^ |  | **√** |  |  | 0.047 | 0.378 | 1.413^*^ |
| 52 | *N*-Acetylglutamine |  | **√** |  |  | 0.033 | 0.370 | 0.324 |
| 53 | *N*-Acetyl-l-aspartic acid | **√** | **√** | **√** | **√** | 0.028 | 0.358 | 1.320^*^ |
|  |  |  |  |  |  | 0.044 | 0.378 | 0.337 |

Supplementary Table S3. The lists of metabolites were identified through 1D and/or 2D NMR spectra (continue).

| No. | Metabolite ID | HMDB | MMCD | HSQC | TOCSY | *p* value | FDR | VIP score |
| --- | --- | --- | --- | --- | --- | --- | --- | --- |
| 54 | *N*-Acetyl-l-tyrosine | **√** |  |  |  | 0.015 | 0.296 | 1.270^*^ |
| 55 | *N*-Acetylneuraminic acid |  | **√** | **√** |  | 0.012 | 0.296 | 1.584^*^ |
| 56 | Niacinamide | **√** |  |  |  | 0.005 | 0.249 | 1.928^*^ |
| 57 | Oxoglutaric acid |  | **√** |  | **√** | 0.044 | 0.378 | 0.337 |
| 58 | Phenylpyruvic acid |  | **√** |  |  | 0.000 | 0.071 | 2.315^*^ |
| 59 | Pipecolic acid |  | **√** |  | **√** | 0.012 | 0.296 | 1.584^*^ |
| 60 | Pyridoxine | **√** |  |  |  | 0.044 | 0.378 | 0.337 |
| 61 | Ribitol |  | **√** |  |  | 0.047 | 0.378 | 1.413^*^ |
| 62 | *S*-Adenosylmethionine |  | **√** |  |  | 0.048 | 0.378 | 1.399^*^ |
| 63 | Salicylic acid |  | **√** |  |  | 0.002 | 0.136 | 2.151^*^ |
| 64 | Salicyluric acid | **√** |  | **√** |  | 0.000 | 0.071 | 2.315^*^ |
| 65 | Tryptamine | **√** |  |  |  | 0.033 | 0.370 | 1.853^*^ |
| 66 | Tyrosol |  | **√** |  |  | 0.047 | 0.378 | 1.413^*^ |
| 67 | Uridine | **√** | **√** |  |  | 0.047 | 0.378 | 1.413^*^ |
| 68 | Valeric acid | **√** |  |  |  | 0.020 | 0.341 | 1.725^*^ |
|  |  |  |  |  |  | 0.012 | 0.296 | 1.584^*^ |

^a^Metabolites were further quantified by LC/MS/MS or HPLC method.^*^Variable importance in projection (VIP) score greater than 1 were used to identify the potential biomarkers. Human Metabolome Database (HMDB); Madison Metabolomics Consortium Database (MMCD); Heteronuclear single quantum coherence spectroscopy (HSQC); TOtal Correlated SpectroscopY (TOCSY)

Supplementary Table S4. Precision and accuracy of determination of 5-aminolevulinica acid in rat urine using HPLC with fluorescent detector (n = 5).

|  | Precision (CV, %) | |  |  | Precision (CV, %) | |  | Accuracy (%) | |
| --- | --- | --- | --- | --- | --- | --- | --- | --- | --- |
| *Standard curve*  *(ng/mL)* | Intra-assay | Inter-assay |  | *Urine* | Intra-assay | Inter-assay |  | Intra-assay | Inter-assay |
| 50 | 8.6 | 8.6 |  | 0 | 1.5 | 4.8 |  | - | - |
| 100 | 7.4 | 7.4 |  | Add 50 ng/mL | 1.6 | 1.4 |  | 103.1 ± 1.5 | 107.8 ± 4.0 |
| 200 | 7.9 | 7.9 |  | Add 100 ng/mL | 1.2 | 3.8 |  | 106.2 ± 0.9 | 109.3 ± 5.0 |
| 300 | 8.3 | 8.3 |  | Add 150 ng/mL | 3.3 | 3.1 |  | 110.4 ± 3.6 | 110.4 ± 4.4 |
| 600 | 4.8 | 4.8 |  |  |  |  |  |  |  |
| 1200 | 6.0 | 6.0 |  |  |  |  |  |  |  |
| 2400 | 4.2 | 4.2 |  |  |  |  |  |  |  |

Supplementary Table S5. Precision and accuracy of determination of guanidinoacetic acid, indoleacetic acid, and hippuric acid in standard solution using LC-MS/MS (n = 3).

| GAA | Precision (CV, %) | | HA | Precision (CV, %) | | IAA | Precision (CV, %) | |
| --- | --- | --- | --- | --- | --- | --- | --- | --- |
| *Standard curve*  *(μg/mL)* | Intra-assay | Inter-assay | *Standard curve*  *(μg/mL)* | Intra-assay | Inter-assay | *Standard curve*  *(μg/mL)* | Intra-assay | Inter-assay |
| 0.05 | 3.1 | 8.5 | 1.25 | 3.5 | 9.5 | 0.0625 | 4.9 | 9.4 |
| 0.2 | 2.3 | 8.5 | 5 | 4.8 | 9.3 | 0.25 | 4.2 | 7.9 |
| 0.4 | 2.6 | 5.5 | 10 | 4.7 | 4.5 | 0.5 | 5.2 | 2.9 |
| 0.8 | 1.3 | 8.0 | 20 | 3.2 | 7.7 | 1 | 4.4 | 7.5 |
| 1.6 | 0.9 | 8.7 | 40 | 3.4 | 9.3 | 2 | 5.7 | 5.8 |
| 3.2 | 5.0 | 6.3 | 80 | 6.5 | 7.9 | 4 | 8.0 | 8.1 |
| 6.4 | 4.7 | 4.5 | 160 | 5.5 | 7.0 | 8 | 4.5 | 5.5 |

Liquid chromatography coupled with tandem-mass spectrometry (LC-MS/MS); guanidinoacetic acid (GAA); hippuric acid (HA); indoleacetic acid (IAA)

Supplementary Table S6. Precision, accuracy, recovery and matrix effect for determination of guanidinoacetic acid, indoleacetic acid, and hippuric acid in rat urine using LC-MS/MS (n = 3).

|  | Recovery | | | | Matrix effect | | | |
| --- | --- | --- | --- | --- | --- | --- | --- | --- |
|  | Precision (CV, %) | | Accuracy (%) | | Precision (CV, %) | | Accuracy (%) | |
|  | Intra-assay | Inter-assay | Intra-assay | Inter-assay | Intra-assay | Inter-assay | Intra-assay | Inter-assay |
| *GAA* |  |  |  |  |  |  |  |  |
| 0 | 4.4 | 1.1 | - | - | 5.7 | 5.4 | - | - |
| Add 0.25 μg/mL | 6.8 | 2.2 | 77.0 ± 5.8 | 74.6 ± 1.2 | 4.3 | 4.0 | 77.2 ± 0.6 | 77.1 ± 2.8 |
| Add 0.5 μg/mL | 3.6 | 2.5 | 61.6 ± 1.1 | 61.7 ± 1.2 | 4.8 | 7.8 | 62.6 ± 1.3 | 61.9 ± 4.9 |
| Add 1 μg/mL | 2.8 | 1.1 | 51.2 ± 0.7 | 51.3 ± 0.7 | 6.9 | 9.1 | 43.0 ± 2.2 | 50.3 ± 5.2 |
| *HA* |  |  |  |  |  |  |  |  |
| 0 | 3.3 | 0.4 | - | - | 4.1 | 3.1 | - | - |
| Add 20 μg/mL | 4.9 | 2.2 | 102.0 ± 3.3 | 100.3 ± 2.1 | 2.2 | 3.6 | 101.6 ± 0.2 | 96.7 ± 2.1 |
| Add 40 μg/mL | 4.2 | 3.5 | 101.5 ± 3.2 | 99.2 ± 3.4 | 4.3 | 8.9 | 101.1 ± 3.0 | 93.5 ± 7.5 |
| Add 80 μg/mL | 3.2 | 3.2 | 98.9 ± 2.4 | 95.7 ± 3.1 | 5.1 | 11.6 | 81.9 ± 3.5 | 93.2 ± 11.3 |
| *IAA* |  |  |  |  |  |  |  |  |
| 0 | 3.1 | 1.1 | - | - | 2.5 | 1.5 | - | - |
| Add 0.25 μg/mL | 5.7 | 3.4 | 102.5 ± 6.0 | 98.8 ± 3.7 | 2.9 | 3.8 | 100.0 ± 1.6 | 95.1 ± 3.6 |
| Add 0.5 μg/mL | 5.0 | 4.0 | 98.8 ± 4.1 | 97.1 ± 4.0 | 5.4 | 7.4 | 99.2 ± 4.6 | 93.1 ± 6.9 |
| Add 1 μg/mL | 3.0 | 1.9 | 98.4 ± 2.4 | 95.2 ± 1.9 | 5.1 | 11.7 | 81.9 ± 3.7 | 93.5 ± 11.0 |
